# Non-traumatic bilateral subdural hematoma in a 1-year-old child with optic glioma: a rare case report and literature review

**DOI:** 10.1097/MS9.0000000000005350

**Published:** 2026-07-24

**Authors:** Solay Farhat, Ali Berro, Mohammad Yazbeck, Nada Sbeiti

**Affiliations:** a Faculty of Medical Sciences, Lebanese University, Beirut, Lebanon; b Department of Pediatric Hematology and Oncology, Lebanese University, Faculty of Medical Sciences, Beirut, Lebanon; c Department of Neurosurgical Sciences, Lebanese University, Faculty of Medical Sciences, Beirut, Lebanon; d Department of Neurosurgery, Al Zahraa University Medical Center, Beirut, Lebanon; e Department of Pediatrics Hematology and Oncology, Al Zahraa University Medical Center, Beirut, Lebanon

**Keywords:** bilateral subdural hematoma, case report, increased head circumference, optic nerve glioma, pediatric oncology

## Abstract

**Introduction and importance:**

Subdural hematoma (SDH) is an uncommon but potentially life-threatening condition in pediatric patients, most frequently associated with trauma or coagulation disorders. Non-traumatic bilateral SDHs are particularly rare, especially in children undergoing treatment for central nervous system tumors. Early recognition is critical, as delayed diagnosis may result in significant morbidity and mortality.

**Case presentation:**

We report the case of a 1-year-3-month-old male with a known optic glioma receiving chemotherapy, who presented with progressive head circumference enlargement (from 56 cm to 59 cm), somnolence, reduced oral intake, and a sunset eye sign. There was no history of trauma or coagulopathy. Brain magnetic resonance imaging revealed large bilateral subacute SDHs with significant mass effect and midline shift. Urgent neurosurgical evacuation with subdural drain placement was performed, yielding over 400 cc of hematoma. The patient demonstrated marked postoperative improvement, with resumption of oral intake within 48 hours and favorable clinical and radiological recovery.

**Clinical discussion:**

While pediatric SDHs are typically trauma-related, this case illustrates a rare non-traumatic presentation in a child with an optic glioma undergoing chemotherapy. The underlying mechanism remains unclear, with possible contributions from tumor-related intracranial pressure changes or treatment-associated vascular fragility. This case highlights the importance of routine surveillance, including serial head circumference monitoring and timely neuroimaging, in pediatric oncology patients.

**Conclusion:**

Non-traumatic bilateral SDHs can occur in children with optic glioma and may present with subtle clinical signs. Early recognition and prompt neurosurgical intervention are essential to achieve favorable outcomes.

## Introduction

Subdural hematoma (SDH) is a serious intracranial condition characterized by the accumulation of blood between the dura mater and the arachnoid mater. It most commonly results from traumatic injury leading to the rupture of bridging veins, although non-traumatic etiologies have been described in association with coagulopathies, vascular abnormalities, and intracranial pathologies^[^1^]^. Based on the time of presentation, SDHs are classified as acute (<72 hours), subacute (3–21 days), or chronic (>3 weeks), with clinical manifestations ranging from acute neurological deterioration to subtle or nonspecific symptoms^[^2,3^]^.

HIGHLIGHTS

- Rapidly progressive bilateral subdural hematomas occurred without trauma or coagulopathy.
- Increasing head circumference was the only early clinical sign.
- Early neurosurgical evacuation led to rapid clinical improvement.
- A possible association between optic glioma, chemotherapy, and spontaneous SDH is suggested.
- Subtle presentations in pediatric oncology patients warrant prompt neuroimaging.

In pediatric populations, SDHs are most frequently associated with trauma, including accidental injury and abusive head trauma. Non-traumatic bilateral SDHs are exceedingly rare, particularly in infants without identifiable risk factors such as bleeding disorders or significant systemic illness^[^4^]^. Their presentation may be atypical, with nonspecific symptoms such as irritability, feeding difficulties, or progressive macrocephaly, which may delay diagnosis.

The occurrence of SDHs in children with intracranial tumors introduces additional diagnostic and therapeutic challenges. These patients may develop intracranial complications in the absence of trauma or overt coagulopathy, and clinical findings may overlap with tumor-related symptoms or treatment effects.

In this report, we describe a rare case of rapidly evolving bilateral subacute SDHs in a 1-year-old child with an optic glioma undergoing chemotherapy. This case underscores the importance of maintaining a high index of suspicion for atypical intracranial complications in pediatric oncology patients and highlights the critical role of early detection and timely intervention.

This case has been reported in accordance with the SCARE 2025 guidelines^[^5^]^.

## Case presentation

**Figure 1.**
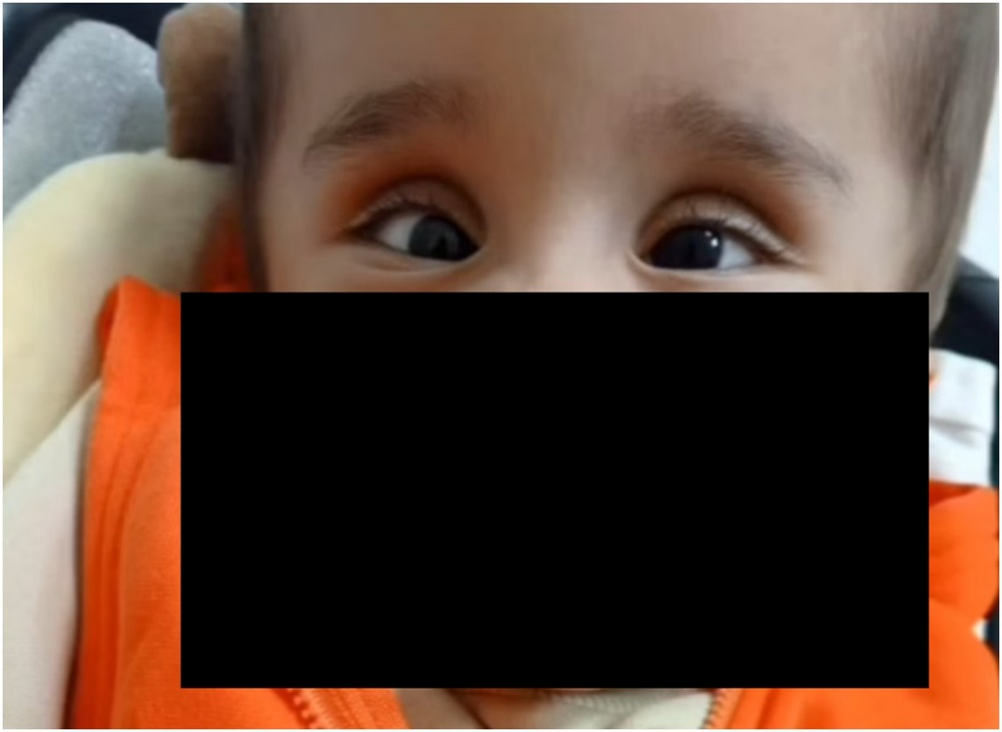
Clinical presentation of the patient’s binocular esotropia.

**Figure 2.**
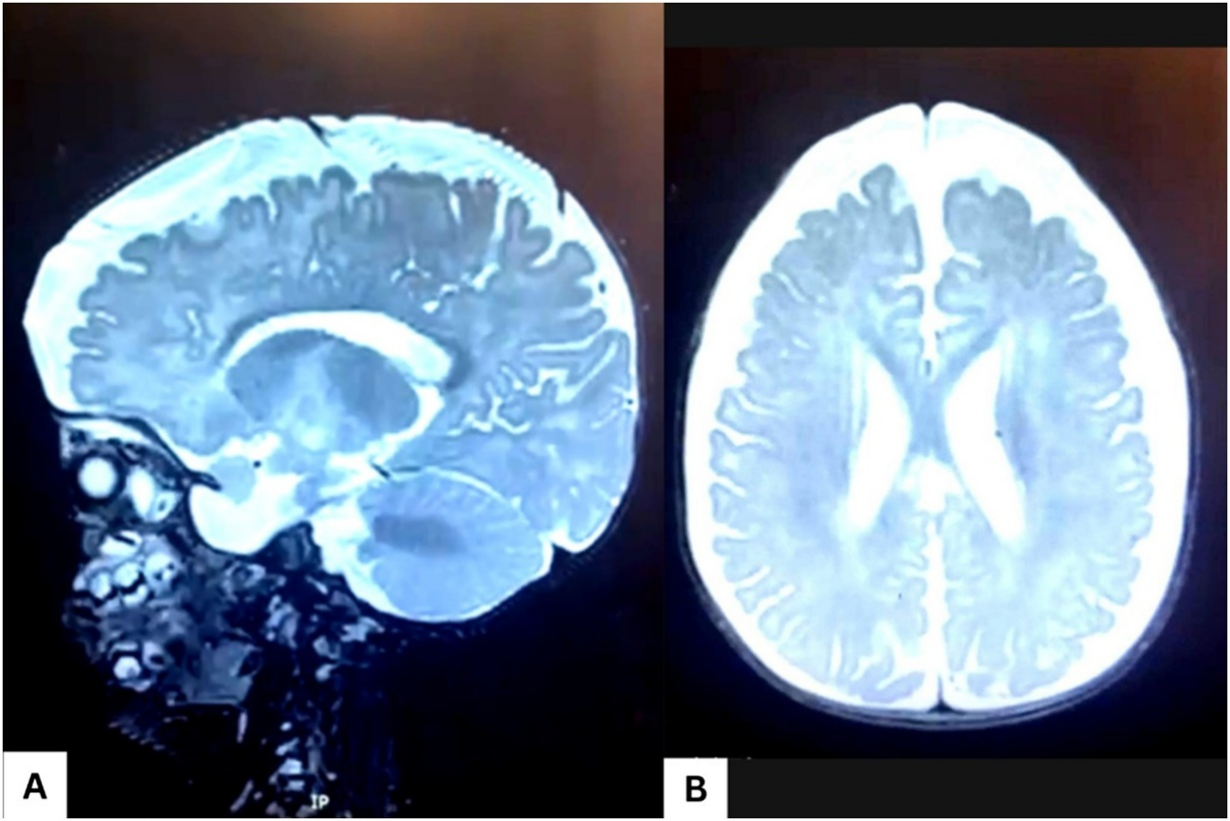
Brain MRI scans at the diagnosis of optic glioma. (A) Sagittal T2 scan; (B) Axial T2 scan.

We present the case of a 1-year-and-3-month-old male, born at term, who was diagnosed with an optic glioma 5 months prior to presentation, following binocular esotropia and progressive head circumference (HC) enlargement (Fig. 1). Initial brain magnetic resonance imaging (MRI) demonstrated an optic pathway glioma measuring approximately 4 × 4 × 3 cm in transverse, anteroposterior, and craniocaudal dimensions. No additional intracranial abnormalities were identified at diagnosis, aside from a minimal subdural hygroma measuring approximately 7 mm over the parietal convexity, without significant mass effect or midline shift (Fig. 2).

The patient was started on chemotherapy and completed 84 days of induction-phase treatment (Supplemental Digital Content Table A, available at: https://links.lww.com/MS9/B308; Supplemental Digital Content Figure S1, available at: https://links.lww.com/MS9/B307). At the initiation of therapy, HC measured 56 cm. He was subsequently referred for a routine follow-up MRI prior to maintenance therapy.

**Figure 3.**
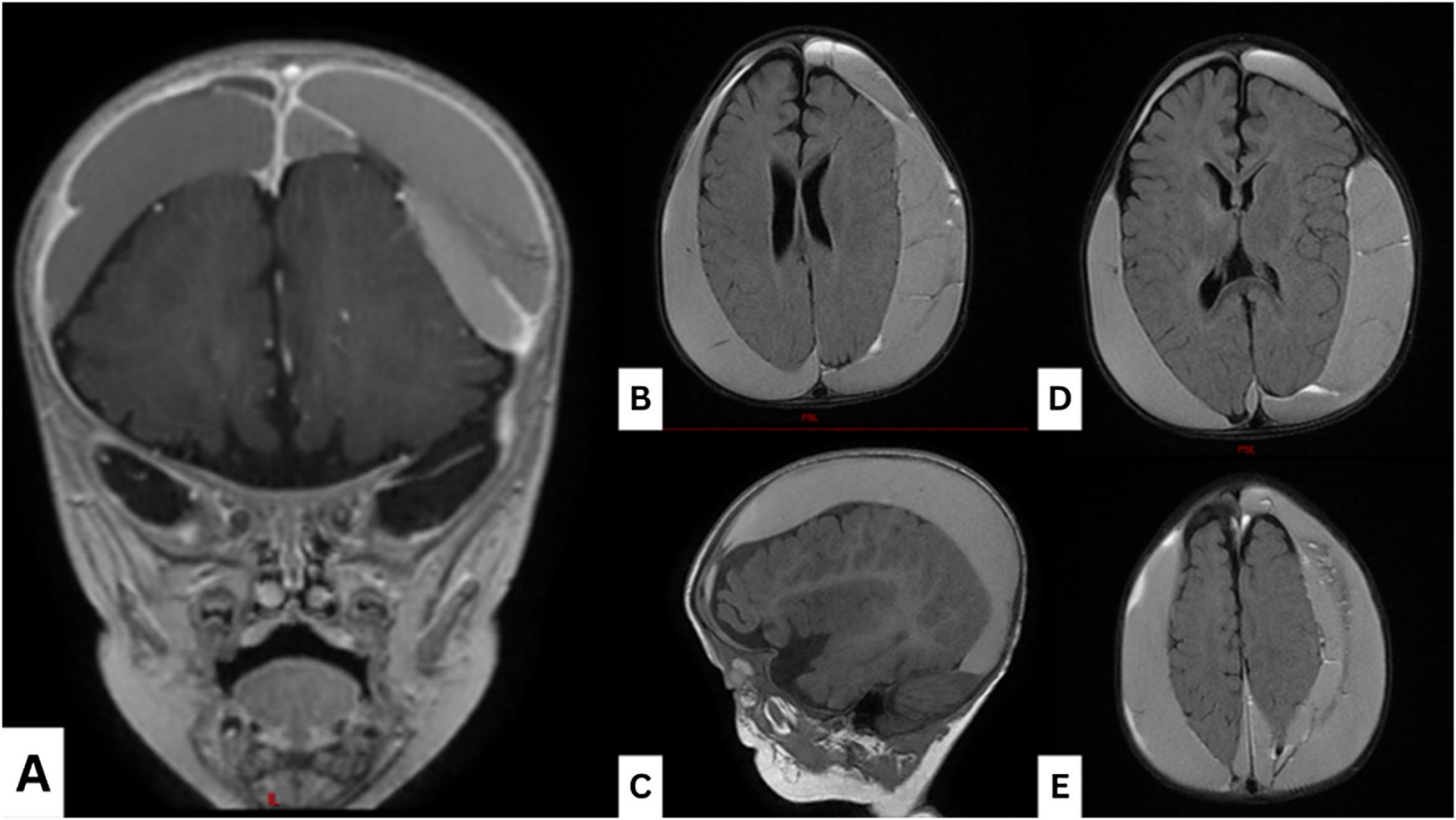
Different MRI cuts showing bilateral subacute subdural hematomas. (A) Coronal cut (top left). (B) Axial cut (top middle). (C) Axial cut (top right). (D) Sagittal cut (bottom middle). (E) Axial cut (bottom right).

Follow-up imaging revealed new and significant findings. A large, early subacute, septated SDH was identified in the left supratentorial region, involving the frontoparietal and temporal areas, with a maximum thickness of 3.6 cm. A similar right-sided SDH, measuring 3.1 cm, was also noted. These collections resulted in significant mass effect with ventricular compression and a mild left-to-right midline shift of approximately 2–3 mm (Fig. 3). Initially, imaging characteristics suggested a possible hemato-hygroma; however, intraoperative findings confirmed the presence of an organized hematoma with multiple septations.

MRI also demonstrated persistent nodular thickening of the optic tracts and chiasm, consistent with the known optic glioma, along with FLAIR hyperintensities along the optic radiations, raising concern for gliomatous infiltration (Supplemental Digital Content Figure S1, available at: https://links.lww.com/MS9/B307).

Clinically, there was no reported history of head trauma. However, the patient developed progressive somnolence, decreased oral intake, and a sunset eye sign. Serial measurements showed further HC enlargement from 56 cm to 59 cm, raising concern for increased intracranial pressure. Laboratory investigations (Table 1) were within normal limits, with no evidence of coagulopathy, infection, or organ dysfunction.

**Table 1:** Laboratory findings of the patient pre-op.

Parameter	Value	Reference range
WBC	4.98 × 10^3^/µL	4.5–11 × 10^3^/µL
Neutrophils (%)	16.4	40–70
ANC	0.82 × 10^3^/µL	>1.5 × 10^3^/µL
Hemoglobin	12.3 g/dL	11.5–14.5 g/dL
Hematocrit	38.2%	35–45%
Platelets	289 × 10^3^/µL	150–400 × 10^3^/µL
Creatinine	0.22 mg/dL	0.2–0.5 mg/dL
PT	12 s	11–14 s
PTT	33 s	25–35 s
INR	1.1	0.8–1.2
CRP	Negative	<5 mg/L
ALT	12.2 IU/L	7–56 IU/L
Total bilirubin	0.2 mg/dL	0.1–1.2 mg/dL
Direct bilirubin	0.11 mg/dL	0.0–0.3 mg/dL

ANC, absolute neutrophil count; CRP, C-reactive protein; INR, international normalized ratio; PT, prothrombin time; PTT, partial thromboplastin time; WBC, white blood cells count.

Given the significant mass effect and clinical deterioration, urgent neurosurgical intervention was undertaken. Bilateral evacuation of the SDHs was performed, with placement of two subdural drains yielding approximately 205 cc and 225 cc of hematoma, respectively. The drains were subsequently removed without complication.

Postoperatively, the patient was monitored in the pediatric intensive care unit for 24 hours. His clinical course was favorable, with only two brief episodes of vomiting that resolved spontaneously within 72 hours. No seizures or neurological deficits were observed. The patient resumed adequate oral intake within 48 hours and demonstrated progressive clinical improvement.

**Figure 4.**
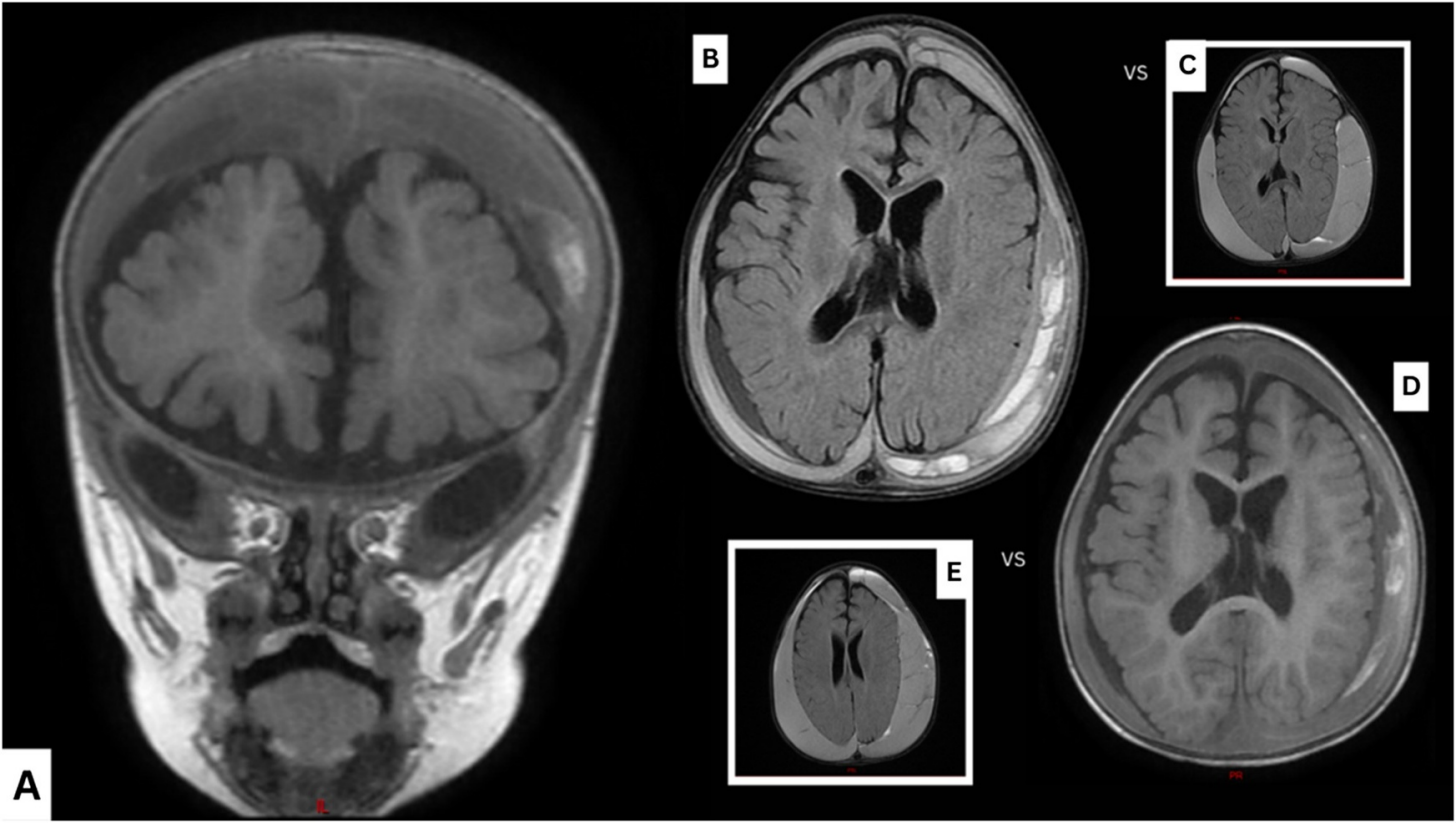
(A) Coronal section showing postoperative changes. (B and D) Axial postoperative MRIs demonstrate improved ventricular size and reduced mass effect compared to the preoperative scans (C and E) at corresponding levels.

Follow-up computed tomography imaging showed stable postoperative findings. A subsequent MRI performed 1.5 months later demonstrated a marked reduction in the size of the bilateral hematomas, with residual subacute-to-chronic collections and a persistent mild midline shift (2–3 mm). Diffuse cerebral atrophy was noted, with no evidence of new ischemia or hemorrhage (Fig. 4).

Clinically, the patient continued to improve, with normalization of feeding and activity levels. HC decreased to 58 cm, and chemotherapy was resumed without further complications.

## Discussion

SDHs are most commonly caused by the rupture of bridging veins due to trauma or rapid intracranial pressure shifts^[^2^]^. Chronic SDHs, in particular, are associated with inflammatory cascades and the formation of vascularized neomembranes that predispose them to recurrent microhemorrhages^[^3^]^.

Epidemiologically, SDHs are more frequently observed in vulnerable populations, including neonates, older adults, and individuals with coagulopathies or those on anticoagulant therapy^[^2,4^]^. In infants younger than 1 year, the incidence ranges between 12 and 24 cases per 100 000, with a peak in early infancy^[^6,7^]^. The majority of cases in this age group are trauma related, including abusive head trauma, which accounts for a substantial proportion of reported cases^[^7,8^]^. Comprehensive reviews suggest an overall incidence of around 16.5 cases per 100 000 in infants, highlighting that earlier estimates may underestimate the true occurrence of SDH in this vulnerable population^[^9,10^]^. In contrast, the present case highlights an exceptionally rare non-traumatic bilateral SDH in an infant, emphasizing the need to consider alternative etiologies in the absence of trauma or coagulopathy.

Bilateral SDHs may result from diffuse intracranial processes, including global pressure alterations or vascular fragility. Clinical presentation varies depending on hematoma size and location, ranging from seizures and lethargy to subtle signs such as feeding difficulties or irritability. Notably, in infants, progressive HC enlargement may represent an early and sometimes isolated manifestation of intracranial pathology due to the compensatory expansion permitted by open cranial sutures. The infant skull has a remarkable capacity to accommodate increases in intracranial volume, which may delay the onset of overt neurological symptoms^[^11,12^]^. This compensatory mechanism likely contributed to the delayed clinical recognition in our patient, where HC progression preceded significant neurological deterioration.

Several studies have reported associations between macrocephaly and SDH in infants. Javadov *et al* described a case of chronic SDH associated with cerebral atrophy and progressive HC enlargement in the absence of trauma^[^13^]^. Similarly, McKeag *et al* reported cases of SDH in infants with benign enlargement of subarachnoid spaces, some presenting primarily with macrocephaly^[^11^]^. Their cohort study identified four cases of SDH out of 177 children with enlarged spaces, some of whom presented with macrocephaly^[^11^]^. These findings support the concept that increased HC should not be overlooked as a benign or developmental finding, particularly in high-risk pediatric populations.

The presence of an underlying optic glioma in our patient adds further complexity. Optic pathway gliomas are low-grade astrocytic tumors that primarily affect the optic nerves, chiasm, and tracts and are frequently associated with neurofibromatosis type 1^[^14,15^]^. While optic gliomas are not classically associated with SDH formation, their presence may indirectly contribute through mechanisms such as increased intracranial pressure or alterations in cerebrospinal fluid dynamics.

Clinically, optic gliomas manifest as visual disturbances, proptosis, or endocrine dysfunction in cases of hypothalamic involvement^[^16^]^. In our case, the patient initially presented with an increased HC relative to his age, with an average of 56 cm at the time of diagnosis (compared to ~43 cm, the average normal HC for a 6-month-old male infant). However, routine HC measurements revealed a minor increase in HC, up to 58 cm, by the time of the scheduled routine MRI. Consequently, the absence of any other neurological deficits led to a low suspicion of any other possible associated pathological incidence. The patient also demonstrated binocular esotropia at the time of the optic glioma diagnosis, and thus its persistence was expected throughout the course of the condition (see Fig. 1).

Additionally, chemotherapy may play a contributory role. Treatment-related vascular fragility, platelet dysfunction, or subtle coagulation disturbances not detected on routine laboratory testing may predispose individuals to spontaneous bleeding events. To our knowledge, except for an abstract published by Mori *et al* in 1975^[^17^]^, there are no documented cases directly linking optic gliomas to SDHs, making this report a valuable contribution to understanding the broader complications of optic pathway gliomas.

Indeed, genetic testing was performed to rule out any underlying hereditary bleeding disorders and revealed an interstitial loss (heterozygous deletion) of approximately 1.6 Mb within the 15q13.2q13.3 chromosomal cytoband (copy number state: 1, classification: pathogenic). The next-generation sequencing finding was confirmed by an orthogonal method chromosomal microarray analysis. This copy number variant encompasses the critical region associated with 15q13.3 deletion syndrome (BP4-BP5). The 15q13.3 microdeletion is a recurrent pathogenic copy number variant associated with a wide spectrum of neurodevelopmental and neuropsychiatric phenotypes, most frequently intellectual disability, developmental delay, epilepsy, autism spectrum disorder, and behavioral abnormalities^[^18–20^]^. Seizures or abnormal electroencephalography findings have been reported in 28–70% of affected individuals, highlighting the syndrome’s strong epileptogenic potential^[^20–22^]^. Dysmorphic features are variably present, including hypertelorism, everted lips, and digital anomalies, but are not consistent across all carriers (Ben-Shachar *et al*, 2009; Van Bon *et al*, 2009). Importantly, penetrance is incomplete, with some carriers exhibiting normal cognitive and neurologic development, suggesting that additional genetic or environmental factors may modulate the phenotype^[^19,23^]^. More recently, large cohort studies have confirmed significant associations between 15q13.3 deletions and neuropsychiatric diagnoses, including schizophrenia and mood disorders, underscoring its pleiotropic clinical consequences^[^24,25^]^. In the context of our patient, the presence of this pathogenic deletion may predispose to vascular or neuronal fragility, thereby potentially contributing to the development of SDHs; however, no literature to date has noted any association between such a genetic deletion and any abnormal bleeding event.

As for the management of SDHs, treatment plans depend on the size of SDHs, chronicity, and clinical presentation. Small, asymptomatic hematomas are often managed conservatively with close monitoring and serial imaging. Surgical evacuation is indicated for symptomatic or large SDHs causing mass effect or midline shift, as in this case^[^26^]^. Postoperative monitoring is essential to detect complications such as recurrence or neurological deterioration.

This case highlights three key clinical challenges: (1) the atypical, non-traumatic presentation of SDH in infancy, (2) the masking of symptoms due to compensatory cranial expansion, and (3) the potential, yet unclear, association with underlying malignancy and its treatment. Importantly, it reinforces the need for a high index of suspicion in pediatric oncology patients presenting with subtle clinical changes. Routine monitoring of HC, combined with timely neuroimaging, may facilitate early detection and prevent life-threatening complications.

Take-home message:

Progressive HC enlargement in infants, even in the absence of trauma or overt neurological deficits, should prompt early neuroimaging, particularly in pediatric oncology patients, where subtle signs may mask serious intracranial pathology.

## Limitations

This case report has several limitations. First, as a single-case observation, causality between optic glioma, chemotherapy, and SDH formation cannot be established. Second, the exact pathophysiological mechanism underlying the development of bilateral SDHs remains speculative, especially in the absence of any associated trauma or underlying bleeding disorders. Finally, despite extensive evaluation, unrecognized contributing factors cannot be entirely excluded.

## Conclusion

This case report describes a rare occurrence of non-traumatic bilateral SDHs in a pediatric patient with an optic glioma undergoing chemotherapy. The progressive increase in HC, initially attributed to normal growth, served as a critical early indicator of intracranial pathology.

Although the exact etiology remains unclear, this case underscores the importance of maintaining a high index of suspicion in pediatric oncology patients presenting with subtle clinical changes. Routine monitoring of HC and timely neuroimaging are essential for early detection. Prompt neurosurgical intervention can lead to favorable outcomes, even in complex presentations.

Further studies are needed to explore the potential relationship between optic gliomas, chemotherapy, and spontaneous SDH formation.

## Data Availability

The data supporting the findings of this study are available from the corresponding author upon reasonable request.
